# Complexity of fractal dimension patterns and machine learning-based classification of altered motor cortical oscillatory activity in rodent models of Parkinson disease

**DOI:** 10.3389/fneur.2026.1816020

**Published:** 2026-05-20

**Authors:** Mesbah Alam, Arif Abdulbaki, Adrian Armstrong, Kerstin Schwabe, Joachim K. Krauss

**Affiliations:** 1 Department of Neurosurgery, Hannover Medical School, Hannover, Germany; 2 Department of Experimental Otology, Institute of AudioNeuroTechnology, Clinics of Otolaryngology, Hannover Medical School, Hannover, Germany

**Keywords:** deep brain stimulation, fractal dimension, Parkinson disease, subthalamic nucleus, support vector machine

## Abstract

**Background:**

In Parkinson disease (PD), enhanced beta frequency band activity in cortico-basal ganglia networks has been proposed as a possible biomarker for adaptive deep brain stimulation (DBS). Previous studies demonstrated enhanced beta frequency peaks both in the acute Haloperidol (HALO) and the chronic 6-hydroxydopamine (6-OHDA) rat models of parkinsonism. Beta peaks decreased after apomorphine (APO) injection or with DBS of the subthalamic nucleus (STN).

**Objective:**

We investigate changes in motor cortical oscillatory activity using fractal dimension (FD) in the HALO and the 6-OHDA rat models of PD. Additionally, we test a support vector machine (SVM) model to predict neuronal dynamics in the 6-OHDA PD model which has been used earlier in an acute rat model of PD.

**Methods:**

In the HALO model, electrocorticogram (ECoG) was recorded from the motor cortex (MCtx) (1) during basal activity, (2) after injection of HALO (0.5 mg/kg), and (3) after subsequent APO injection (1 mg/kg). For the chronic 6-OHDA model, MCtx-ECoG recordings were obtained (1) during basal activity, and (2) during STN DBS. Higuchi's FD algorithm and SVM-based classification were utilized for analysis.

**Results:**

Average FD values in the MCtx were higher in both PD models compared to controls (*P* < 0.001). APO injection (*P* < 0.001) and STN DBS (*P* < 0.05) reduced average FD values in both models. The SVM model achieved 80% classification accuracy and an AUC of 0.86 in the 6-OHDA rat model.

**Conclusion:**

The non-linear analysis of FD reveals changes in cortical oscillatory patterns in rodent models of PD. SVM-based predictions demonstrate potential for classifying altered neural activity, which may offer future strategies for adaptive DBS.

## 1 Introduction

Parkinson disease (PD) is characterized by degeneration of dopaminergic neurons in the substantia nigra, which leads to dopamine (DA) deficiency in the nigrostriatal system and subsequent dysfunction of the motor cortico-basal ganglia circuitry. These network abnormalities are associated with increased beta-band synchronization and altered neuronal firing patterns (1, 2).

In the last two decades, substantial effort has been devoted to identifying and understanding beta-band LFP biomarkers in PD and their potential application in clinical practice (3, 4). Increased beta-band oscillations (12–30 Hz) and enhanced beta coherence in the cortico-basal ganglia network are considered electrophysiological hallmarks of PD and correlate with the severity of motor symptoms, including hypokinesia, rigidity, and bradykinesia (5). Clinical and experimental studies have shown that both dopaminergic treatment and deep brain stimulation (DBS) of the subthalamic nucleus (STN) reduce exaggerated beta-band oscillations in the basal ganglia and sensorimotor cortex, accompanied by improvement in motor symptoms in patients with PD (6–8) or in rodent models of Parkinsonism (9–11). Clinical studies in PD have suggested that the duration of beta episodes and the occurrence of beta bursts may represent more specific and robust indicators of parkinsonism than mean beta power alone (12, 13). Nevertheless, beta oscillations are not inherently pathological, as they also occur in various brain regions of healthy individuals (14, 15).

Altered patterns of neural oscillatory activity, occurring both within single frequency bands and in the relationships between different frequency bands, are thought to contribute to motor dysfunction in PD (16). These complex changes may also hold potential as biomarkers for closed-loop DBS approaches (17, 18). Recently, there has been significant interest in extracting non-linear features in brain signals in order to understand the dynamics of basal ganglia circuitry and to interpret the neuronal dysfunctions underlying parkinsonian motor symptoms (19–23). The geometric complexity or irregularity of patterns in data can be quantified by fractal dimension (FD), which has been proposed as a useful tool for characterizing the complexity of brain activity (24). Furthermore, clinical studies have shown that Higuchi'sfractal dimension (HFD)is sensitive to brain activity changes observed in healthy aging, PD and Alzheimer's disease (25–27). This suggests that HFD analysis may provide information beyond conventional spectral measures and may help to capture aspects of cortical dysfunction that are not reflected by beta power alone.

The present work builds on two of our previously published studies in experimental parkinsonism. In the first, we investigated the acute haloperidol (HALO) induced rat model and analyzed oscillatory activity across different frequency bands together with deep learning-based classification using a convolutional neural network (28). In the second, we examined the chronic 6-hydroxydopamine (6-OHDA) lesion model and showed pathological alterations not only in the beta band, but also in theta, alpha, and gamma oscillations, although machine learning-based classification was not included in that study (2). The present study extends these findings by combining non-linear FD analysis in both models with support vector machine (SVM)-based classification in the chronic 6-OHDA model. This approach allows us to examine whether dopamine depletion affects not only spectral activity, but also the temporal complexity of motor cortical network dynamics, and whether these altered dynamics can be computationally identified in a chronic lesion-based model of parkinsonism.

While these previous studies identified spectral abnormalities in acute and chronic Parkinsonian rat models, they did not address whether these states are also associated with altered temporal complexity of cortical signals. We hypothesized that DA loss in PD alters not only oscillatory activity within individual frequency bands but also the temporal dynamics, or synergy, of their modulation in motor cortical output networks. Specifically, we investigated non-linear changes in HFD in the motor cortex in an acute and in a chronic rat model of PD with DA depletion induced by administration of the neurotoxin into the medial forebrain bundle, thereby lesioning the nigrostriatal pathway. We further examined whether antiparkinsonian treatment with apomorphine (APO) in the acute HALO model and STN-DBS in the chronic 6-OHDA model modify these FD changes. Therefore, in the present study HFD analysis was performed in both the acute HALO model and the chronic 6-OHDA model, enabling comparison of cortical dynamics across these two Parkinsonian states. Further, changes in cortical FD values were evaluated after anti parkinsonian treatment of apomorphine and STN-DBS in acute and chronic rat model of PD. In addition, SVM analysis was applied in the chronic 6-OHDA model to test whether altered cortical patterns could be predicted computationally. By moving beyond conventional spectral analysis, this approach extends our previous work toward a more integrative characterization of Parkinsonian cortical dynamics and evaluates FD as a potential additional biomarker of pathological brain activity.FD analysis may provide additional insight into the effects of dopamine depletion beyond conventional spectral activity measures, particularly regarding the non-linear complexity of cortical network states.

## 2 Materials and methods

### 2.1 Ethics and re-analysis of curated ECoG data

We analyzed motor-cortical ECoG recordings from previously published acute and chronic rat models of PD (2, 28). Here, we re-examined these datasets using an alternative analysis framework to provide further insights beyond the original work. All experimental procedures for these studies were approved by the responsible local authority (permit no. AZ 16/2313) and conducted in accordance with the German Animal Welfare Act (Tierschutzgesetz) and institutional guidelines, as detailed in the original publications.

In this secondary analysis, we re-used the raw ECoG data and applied a non-linear analytical approach (FD-based feature extraction combined with SVM-based classification) to extend the original findings, in line with ethical principles and the 3R concept, particularly Reduction, without any additional animal experimentation.

### 2.2 Data source and study design

Detailed descriptions of the animal surgical studies have been reported in our previous studies (2, 28). In brief, data sets of two different rat models of PD were used:Acute HALO-induced rat model of PD and chronic 6-OHDA-inducedrat model of PD.

Six male Sprague–Dawley rats (260–350 g; Charles River Germany) were used to induce an acute Parkinsonian state via systemic administration of the dopamine receptor antagonist HALO (0.5 mg/kg, s.c.). An ECoG electrode was implanted over the motor cortex (MCtx; *n* = 6). On the sixth recovery day after implantation surgery, three ECoG recording sessions (300 s each) were acquired: (i) baseline activity without pharmacological intervention (control), (ii) after HALO administration (0.5 mg/kg, s.c.) to induce an acute Parkinsonian state, and (iii) post-treatment with apomorphine (APO, 1 mg/kg, s.c.). This was administered to alleviate the HALO-induced Parkinsonian symptoms (28).

For the chronic rat model of PD, rats were injected with 6-OHDA unilaterally into the medial forebrain bundle (*n* = 8), and were classified as hemi-parkinsonian (HP) rats. The sham-lesion (control) rats (*n* = 7) were injected with 0.02% ascorbate saline solution as vehicle to serve as control. Afterwards, ECoG micro-electrode arrays were implanted over the MCtx, followed by the implantation of STN DBS electrodes in the right hemisphere ipsilateral to the 6-OHDA lesion side. On the sixth recovery day, cortical activity was recorded for 300 s under two different conditions: baseline (no stimulation) and during STN DBS (more detailed description in (2, 10, 23).

During recording, rats were placed in a glass cylinder (30 × 28 cm) on a vibration-isolating table inside a Faraday cage to minimizeelectromagnetic interference. The base of the glass cylinder was an aluminum plate that served as grounding for animals. With this experimental setup, motor-cortical ECoG recordings were measured in a freely behaving condition.

In the chronic6-OHDA rat model of PD, HP rats yielded 64 epochs (8 rats × 8 channels), whereas sham-lesion rats yielded 56 epochs (7 rats × 8 channels), resulting in 120 epochs across 15 recording sessions. Baseline recordings were considered to represent the healthy state (sham-lesioned non-PD). Each session lasted 300 s.

Each channel was analyzed individually, such that each sample corresponded to a single electrode from the eight-channel microelectrode array. For each channel, a HFD value was extracted, resulting in an eight-dimensional feature vector per animal. This feature representation was then used as input for the SVM classifier.

### 2.3 Data processing and FD analysis

The ECoG signals were band-pass filtered between 0.5 and 100 Hz and sampled at 1,000 Hz. The raw signals (1 k samples/s) were saved in the .ns2 digitized data format and subsequently converted into MATLAB data files. The data were down sampled to 256 Hz. Furthermore, Higuchi's FD algorithm analysis was applied on the ECoG data. To analyze the complexity of LFP signals the Higuchi dimension (29) was computed from the spontaneous LFP activity using Matlab as recently described by Stenzel et al. (30). Previous studies have reported that calculating the FD of EEG signals using Higuchi's algorithm is the most appropriate method for analyzing electrophysiological data (31, 32). We selected *k*_max_ at the point where the FD curve reached a stable plateau, ensuring that further increases did not systematically change the FD values.

Given a time series X : { 1, …, N } → ℝ consisting of *N* data points and a parameter *k*_max_≥2 the HFD of *X* is calculated in the following way: For each 1 ≤ *k* ≤ *k*_max_ and 1 ≤ *m* ≤ *k* define


$$\begin{array}{l} L_{m}\left(k\right)=\frac{N-1}{\left\lfloor \frac{N-m}{k}\right\rfloor k^{2}}\overset{\left\lfloor \frac{N-m}{m}\right\rfloor }{\underset{i=1}{\sum }}|X\left(m+ik\right)-X\left(m+\left(i-1\right)k\right)|. \end{array}$$

The HFD is defined as the slope of the best-fitting linear function through the data points $\left(\log\frac{1}{k},\log L\left(k\right)\right),1\leq k\leq k_{\max}$ where $L\left(k\right)=\frac{1}{k}\overset{k}{\underset{m=1}{\sum }}L_{m}\left(k\right).$ We estimated HFD for *k*_max_ = 2, ..., 100.

The Higuchi dimension is between 1 and 2, a higher value corresponding to higher signal complexity.

**Figure 1:**
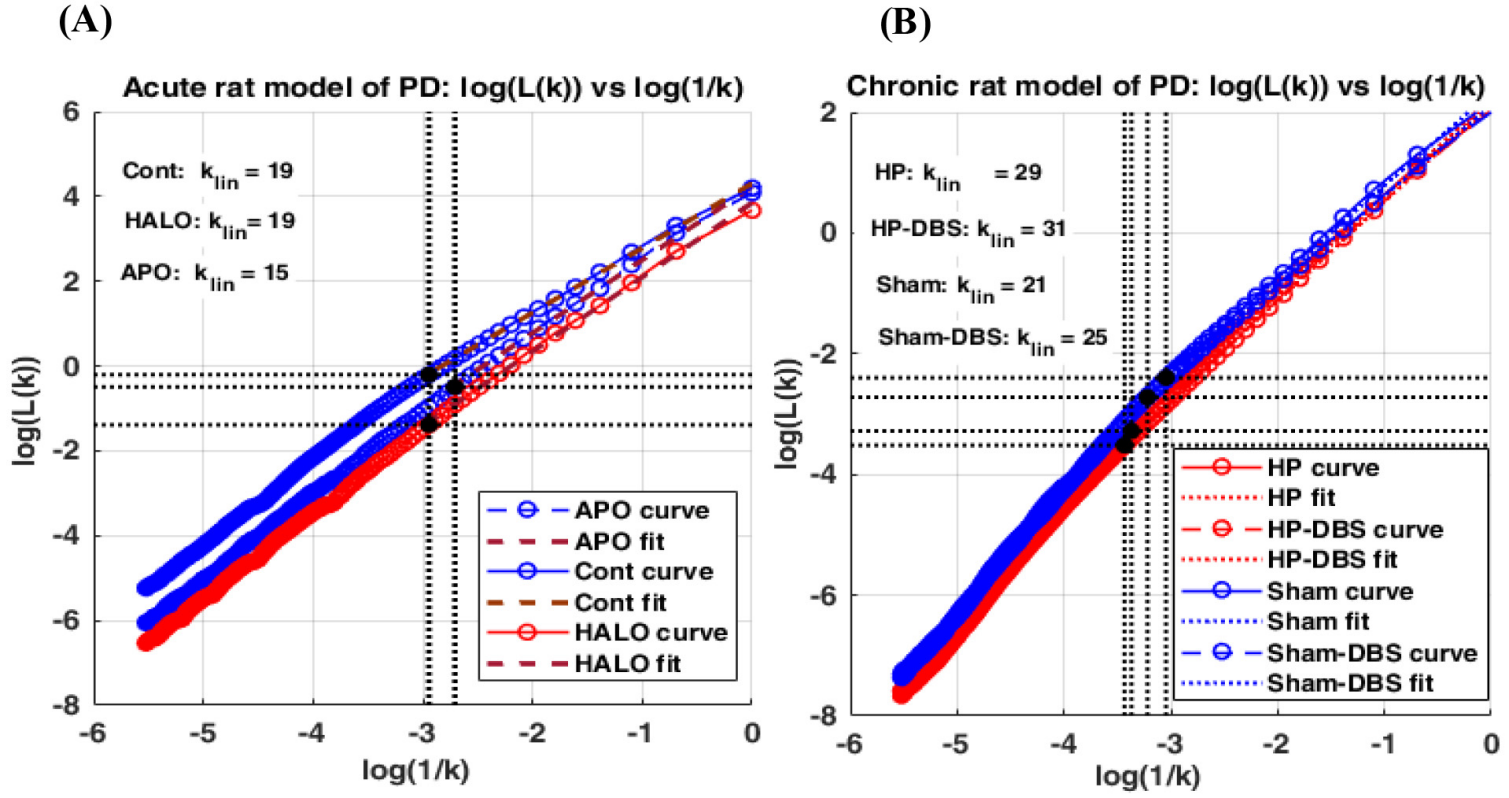
**(A, B)** Log–log plots of log L(*k*) vs. $\log\left(\frac{1}{k}\right)$for the acute and chronic rat models of PD. Group curves are shown for the acute model (Control, HALO, and APO; A) and the chronic model (HP, HP-DBS, Sham, and Sham-DBS); **(B)**. Vertical dotted lines indicate the group-specific *k*_max_ or *k*_lin_ values marking the onset of the linear scaling region. Across groups, stable scaling behavior was observed from *k* = 19 in the acute model and from *k* = 31 in the chronic model. The high linearity of the fitted segments (R^2^ > 0.99) supports the use of these scaling regions for HFD estimation.

The parameter *k*_max_ was determined empirically from the log L(*k*) vs. $\log\left(\frac{1}{k}\right)$ relationship by identifying the onset of the linear scaling region. On a logarithmic scale, this relationship is expected to remain linear over a defined range of *k* values, reflecting the self-similarity of the time series, a key property of fractal structures (33). Restricting the estimation to this linear scaling region provides an objective and mathematically consistent estimate of signal complexity. In the acute rat model of PD, the corresponding *k*_max_ values were 19 for control, 19 for HALO, and 15 for APO (Figure 1A). In the chronic model, the resulting *k*_max_ values were 29 for HP, 31 for HP-DBS, 21 for Sham, and 25 for Sham-DBS (Figure 1 B). These values were used for subsequent HFD estimation in the respective groups. To ensure comparability across groups, a common value was defined for each model based on the upper bound of the group specificestimates, yielding *k*_max_ = 19 for the acute rat model and *k*_max_= 31 for the chronic rat model.

### 2.4 Subject-level classification using support vector machine

Subject-level classification was performed to assess whether channel-levels HFD measures could distinguish PD animals from healthy controls. Each animal was represented by a single feature vector comprising eight HFD values, one from each recording channel. Accordingly, the dataset consisted of 15 independent samples, including 8 PD animals and 7 healthy controls.

#### 2.4.1 Training procedure and cross-validation strategy

Classification was performed using a linear SVM implemented in MATLAB using the fitcsvm function. A linear kernel was selected because of the small sample size and low-dimensional feature space. Within each outer training fold, the regularization parameter (BoxConstraint) was optimized by inner cross-validation across the values 0.1, 1, and 10. The final classifier was then trained on the complete training set of the respective fold and used to predict the class label of the held-out subject.

Model performance was evaluated using leave-one-out cross-validation. In each fold, one animal was excluded from model training and used exclusively for testing, while the remaining animals constituted the training set. This procedure was repeated until every subject had served once as the test sample.

#### 2.4.2 Performance evaluation

The evaluation metrics employed for the final assessment comprised accuracy $\frac{TP+TN}{\left(TP+TN+FP+FN\right),}$ sensitivity $\frac{TP}{\left(TP+FN\right),}$ specificity $\frac{TN}{\left(TN+FP\right)}$ and precision $\frac{TP}{\left(TP+FP\right)}$, where *TP* is the number of true positives, *FN* the number of false negative*s*, *TN* the number of true negatives and *FP* the number of false positives. Model discrimination was additionally quantified using the area under the receiver operating characteristic curve (ROC-AUC). The ROC curve plots the true positive rate against the false positive rate across different decision thresholds, whereas the AUC provides a threshold-independent summary measure of classification performance. Statistical significance of the observed classification accuracy was further assessed using permutation testing.

#### 2.4.3 SVM decision boundary-based visualization

For visualization purposes, a representative pair of features was selected from the full feature set. The corresponding SVM decision boundary was computed in a two-dimensional feature space using a custom svm plot function.

### 2.5 Statistical analysis

A one-way repeated measures analysis of variance (ANOVA) was performed for the acute rat model, while a two-way mixed design repeated measures ANOVA was used for the chronic rat model. Subsequently, the *post-hoc* Fisher's least significant difference (LSD) test was performed to directly compare groups or treatment factors. The Shapiro-Wilk test (normality assumption) and Levene's test for homogeneity of variances yielded non-significant results i.e., greater than (*P* > 0.05) and confirmed that the acceptance criteria were met. Consequently, the repeated measures analysis approach was chosen. *P* < 0.05 was considered statistically significant.

## 3 Results

### 3.1 FD value changes in the acute rat model of PD (baseline, HALO, and APO)

**Figure 2:**
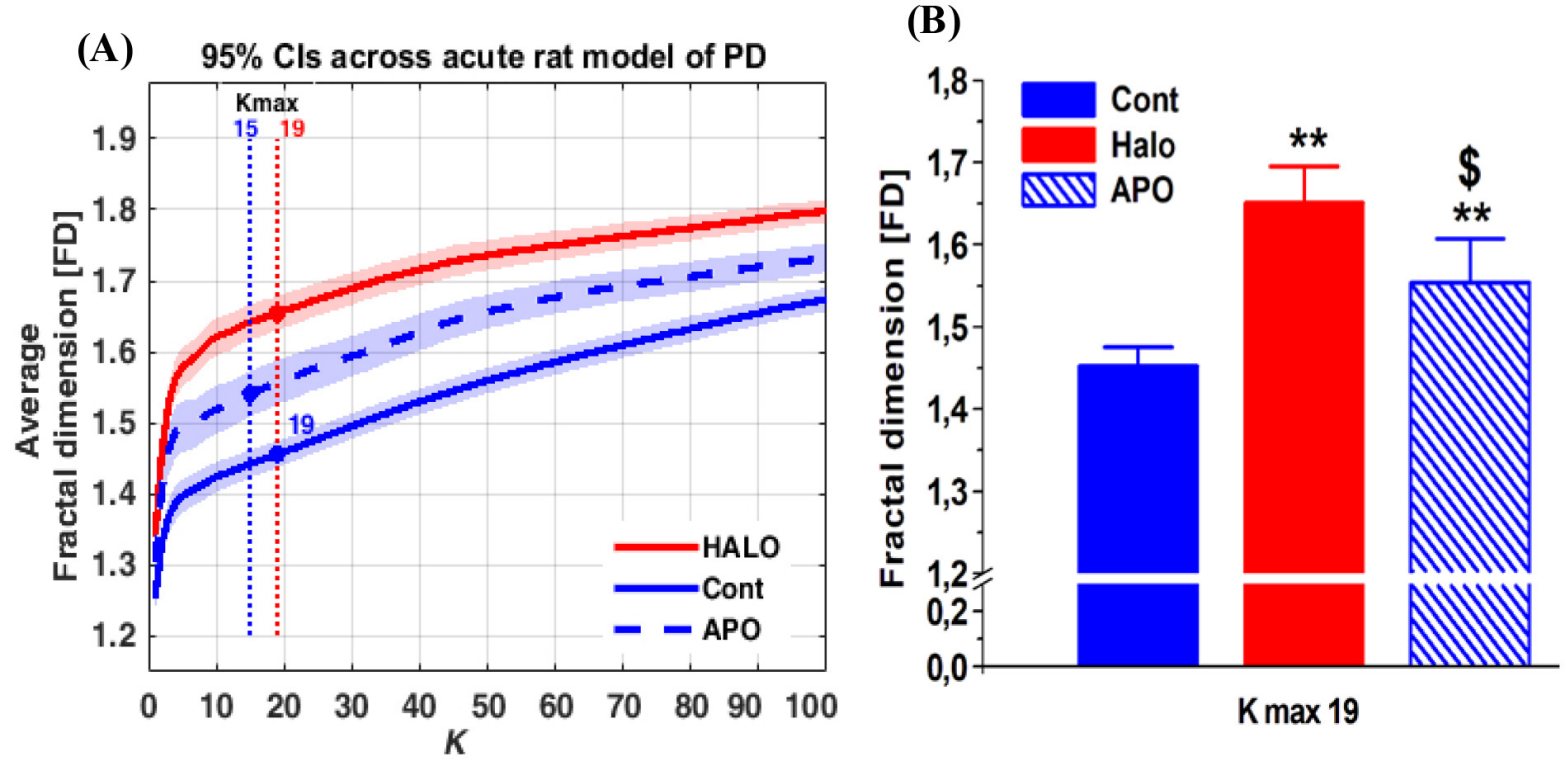
**(A, B)** The average FD of the MCtx oscillatory activity of the original raw ECoG signals of drug free naïve condition (control), after injection of haloperidiol (HALO) and apomorphine (APO) and light shading indicating 95 % confidence intervals. *k*_max_ = 10represents the starting point of FD stability with respect to the difference between groups **(A)**. The selected *k*_max_ = 10 is plotted as bar graphs **(B)** The values are shown as mean and standard error of mean. Significant differences in comparison to naïve condition (control) are indicated by (**P <0.01) and differences between HALO and APO are indicated by ($; *P* < 0.05). One way ANOVA followed by Fisher's LSD test for comparisons of different groups.

**Figure 3:**
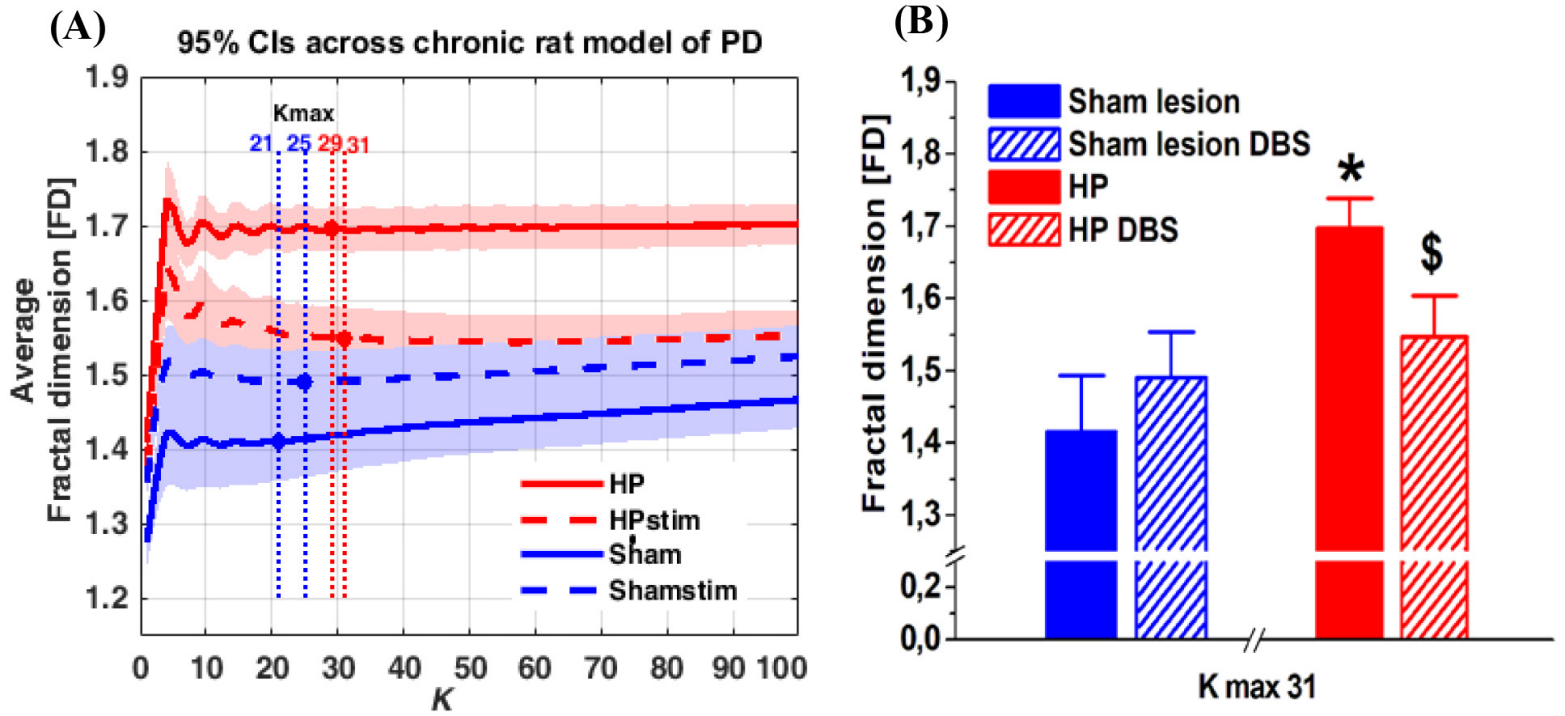
**(A, B)** The average FD of the MCtx oscillatory activity of the original raw ECoG signals of hemi-parkinsonian rats (HP; *n* = 8) and sham-lesion rats (Sham; *n* = 7) before STN DBS (basal activity) and during STN DBS (light shading indicating 95 % confidence intervals). *k*_max_ = 25 represents the starting point of FD stability with respect to the difference between groups **(A)**. The selected *k*_max_ = 25 is plotted as bar graphs **(B)** Significant differences in comparison to sham lesion (control) are indicated by (**P < 0.01) and differences between HP and STN DBS are indicated by ($; *P* < 0.05). Two way ANOVA followed by Fisher's LSD test for comparisons of different groups.

We observed that the FD reached stability at *k*_max_ = 19 (Figure 2A) in the acute rat model and a *k*_max_ = 31 (Figure 3A) in the chronic rat model. We used these specific *k*_max_ values to assess statistically significant differences across the groups.

In the acute rat model, statistical analysis with ANOVA found significant differences across different treatment groups [*F*_(2, 17)_ = 19.45; *P* < 0.001]. *Post-hoc* comparison with Fisher's LSD test revealed that the injection of HALO resulted in a significant increase (*P* < 0.001) in the FD. Conversely, following the injection of APO, the FD values were significantly reduced (*P* < 0.01). Furthermore, notable differences were observed between the control (basal condition) and the FD values after APO injection (*P* < 0.05; Figure 2B).

### 3.2 FD value changes in the chronic rat model of PD and modulation by STN DBS

In the chronic rat model, statistical analysis with ANOVA found significant differences for the factor group [*F*_(1, 13)_ = 5.98; *P* < 0.05]; and differences for the interaction between the factors treatment and groups [*F*_(1, 13)_ = 5.37; *P* < 0.05]. *Post-hoc* comparison of Fisher's LSD test HP rats showed a significant increase (*P* = 0.003) in the FD compared to the control group of sham lesion rats. During STN DBS, the FD values decreased significantly (*P* < 0.05) in the HP rats, whereas STN DBS did not alter the FD for the sham lesion control rats (Figure 3B).

### 3.3 Subject-levels SVM classification performance

**Figure 4:**
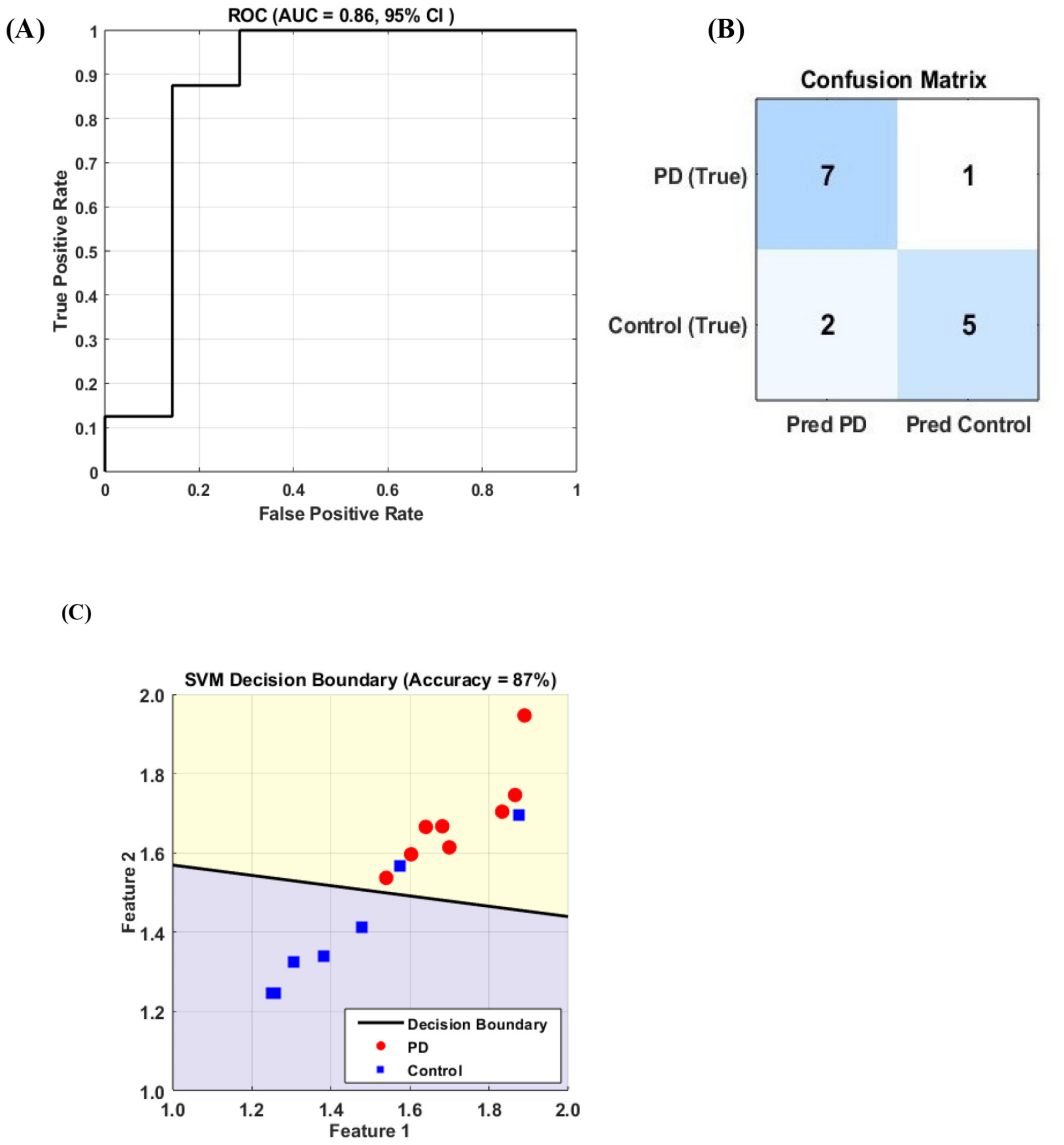
**(A-C)** SVM classification of sham (control) and hemi-parkinsonian (HP) rats based on fractal dimension (FD) features extracted from MCtx-ECoG recordings, showing **(A)** ROC curve and AUC, **(B)** confusion matrix, and **(C)** SVM decision boundary-based visualization in a two-dimensional feature space using a representative pair of features selected from the full feature set. Model performance reached 80.0% accuracy and 87.5% sensitivity.

The linear SVM classified PD and control animals based on HFD features with an accuracy of 80.0%. Sensitivity for PD detection was 87.5%, specificity was 71.4%, and precision was 77.8% with an F1-score of 82.4%. ROC analysis yielded an AUC of approximately 0.86, indicating moderate class separability (Figure 4A). The corresponding confusion matrix is shown in Figure 4B, with 7/8 PD animals correctly classified (7 true positives, 1 false negative) and 5/7 control animals correctly classified (5 true negatives, 2 false positives).

At the subject level, 7 of 8 PD animals and 5 of 7 control animals were correctly classified. These results suggest that HFD features contain relevant group information, although performance should be interpreted cautiously due to the limited sample size.

The selected feature pair was used to visualize the SVM decision boundary in a two-dimensional projection (Figure 4C), illustrating class separability between PD and control animals. This representation is intended solely for visualization and does not reflect the full high-dimensional feature space used for model training. Using this feature pair, the model achieved an accuracy of 80.0% and a sensitivity of 87.5%.

## 4 Discussion

### 4.1 Higher FD values in parkinsonian states which reduced by dopaminergic therapy or DBS

Our results indicate that both the acute and the chronic rat models of parkinsonism are associated with higher FD, suggesting an increase in complexity or irregularity in the ECoG signals within the motor cortical oscillatory activity.

Higher complexity or irregularity in brain signals has been linked to motor symptoms and movement execution in PD patients (20, 34). Previous studies using non-linear analysis of brain neuronal activities have shown that patients with PD exhibit increased entropy or higher irregularity in their brain signals (20, 35). Similarfindings were observed in experimental animal models of PD (23, 36). Moreover, DA replacement therapy or DBS as an intervention approach has been found to reduce entropy or irregularity in patients with PD (37) and also in animal models of PD (37, 38). Our study also found that both the administration of the DA agonist APO and STN DBS led to a significant reduction in FD, suggesting a potential return toward baseline level. These findings suggest that FD reflects biologically meaningful alterations in cortical network state associated with dopaminergic dysfunction and its therapeutic modulation. In a recent clinical study, a comparable finding of increased FD was noted in individuals with PD when compared to healthy controls in electroencephalogram (EEG) signals (26).

New mathematical models are required to characterize and interpret brain signals in order to understand brain function in different pathological brain conditions. Such mathematical models and analysis may help researchers to uncover meaningful patterns and relationships in brain EEG or ECoG data, providing insights into brain function and dysfunction. One potential approach that falls within this domain involves using non-linear analysis models of EEG. This non-invasive technique has the capability to depict brain electrical activity with both high temporal resolution and high test-retest reliability (35, 39, 40). These studies indicate that quantifying FD of brain oscillatory activity may also be useful in describing the pathophysiology of PD.

### 4.2 FD as a non-linear biomarker candidate for PD-related cortical dynamics and implications for aDBS

Non-linear signal analysis is a promising alternative option of dealing with EEG complexity, considering the latter as the result of non-linear deterministic dynamics, possibly representing a chaotic process (41–43). The outcomes of our FD study offer new insights into the biomarker aspects of PD concerning motor cortical areas. These findings also hold significant potential in optimizing the implementation of close-loop DBS development strategies. The FD is a quantitative measure of shape complexity used widely for analyzing of brain oscillatory activity from EEG signals in various neuropsychiatric disorders (44). Decreased values of EEG complexity have also been reported in patients with schizophrenia compared to healthy individuals (26, 45). In neurodevelopmental psychiatric disorders, oscillatory activities differ from those observed in movement disorders like PD (46).

### 4.3 SVM-based discrimination of parkinsonian vs. control activity: practical implications

The subject-level classification results show that channel-wise HFD features carry discriminative information that allows separation of Parkinsonian and control animals. An accuracy of 80.0% and an AUC of 0.86 reflect moderate classification performance, with particularly high sensitivity (87.5%) for identifying PD animals. This indicates that fractal-based features capture meaningful disease-related changes in cortical activity. The lower specificity (71.4%) suggests that control animals were more often misclassified, which may be related to overlapping feature distributions or variability introduced by the small sample size.

We also observed higher FD values in the PD group compared to controls, suggesting increased complexity in cortical activity under Parkinsonian conditions. This finding is consistent with previous reports showing that non-linear measures such as fractal dimension and entropy can distinguish PD from healthy states and achieve strong diagnostic performance, including AUC values up to 0.97 in ROC analyses (26). Although the present study yielded a slightly lower AUC of 0.86, the result remains in a comparable range, particularly considering the smaller sample size and reduced dataset, which are known to limit classifier performance and stability. A small numberof motor cortical of channels (as few as one to eight) were sufficient to achieve good classification performance.

Motor cortical ECoG channels appeared to contribute most strongly to classification, suggesting that motor cortical dynamics are particularly affected in this model. This agrees with established evidence of motor cortex involvement and broader network-level dysfunction in PD and supports the idea that fractal dimension measures capture relevant pathological changes in neural activity. Previous work has shown that non-linear approaches, including entropy and fractal-based measures, are useful for characterizing neurodegenerative changes in PD and may have value as diagnostic and prognostic markers (20, 43, 47, 48).

However, these findings should be interpreted with caution given the small sample size, which limits generalizability and may increase the risk of overfitting despite cross-validation. Larger datasets will be necessary to validate these results and to further explore the spatial specificity of the most informative channels.

A substantial body of research has focused on brain signal-processing pipelines based on handcrafted feature extraction (49–52). Adaptive deep brain stimulation (aDBS) is an important step toward personalized therapy with precise onset of DBS for various neurological disorders, as in particular for PD (53–56). There is a need for robust and reliable hardware and software systems for neural signal acquisition in aDBS which should be carefully addressed with a multidisciplinary approach involving experts in machine learning, neuroscience, clinical practice, ethics, and regulatory affairs. The feasibility and the success of DBS therapy in PD patients will rely on the advancement of approaches to recognize both normal and abnormal patterns of brain signals, along with receiving feedback to the pulse generator to determine the optimal timing for delivering stimulation (18). Progress in these fields would facilitate the accurate application of customized stimulation for each individual patient.

### 4.4 Conclusions and future directions for non-linear biomarkers in aDBS

Overall, this study provides further insight into alterations in brain activity associated with Parkinsonian states and highlights the potential of signal complexity-based approaches for classification. In a previous study using an acute rat model of PD, deep learning-based methods captured electrophysiological features such as beta-band alterations (28). In the present work, an SVM model was used to identify Parkinsonian pathology using fractal dimension features. Several research studies have applied FD and machine learning techniques for the detection of brain abnormalities, demonstrating the usefulness of FD in obtaining biomarkers for neurological and neurodevelopmental disorders (26, 47, 57, 58). A further step would be to integrate FD-based thresholds into an adaptive DBS loop, where stimulation is triggered upon detection of pathological activity patterns.

The FD, which is a non-linear method, enabled clear differentiation of ECoG patterns between conditions. Moreover, the classification performance of the SVM suggests potential applicability for future aDBS systems. Future work should further evaluate the trade-off between computational efficiency and classification performance, with particular focus on optimizing feature-based models for implementation in resource-constrained aDBS hardware. However, these findings remain preliminary and require validation in larger datasets before clinical translation.

### 4.5 Limitations

Overall, while the SVM-based classification using FD features shows promising discriminative potential, further studies with larger cohorts, including sham-lesioned and healthy control groups as well as additional EEG channels, are required to validate its robustness and to support its integration into real-time aDBS systems.

## Data Availability

The data analyzed in this study is subject to the following licenses/restrictions: All data and materials are presented in the article, and additional supporting files are available from the corresponding author upon reasonable request. Requests to access these datasets should be directed to alam.mesbah@mh-hannover.de.
